# Nanocarrier cancer therapeutics with functional stimuli-responsive mechanisms

**DOI:** 10.1186/s12951-022-01364-2

**Published:** 2022-03-24

**Authors:** Neha Kaushik, Shweta B. Borkar, Sondavid K. Nandanwar, Pritam Kumar Panda, Eun Ha Choi, Nagendra Kumar Kaushik

**Affiliations:** 1 Department of Biotechnology, College of Engineering, The University of Suwon, Hwaseong, 18323 Republic of Korea; 2 Department of Electrical and Biological Physics, Plasma Bioscience Research Center, Kwangwoon University, Seoul, 01897 Republic of Korea; 3 Department of Basic Science Research Institute, Pukyong National University, Busan, 48513 Korea; 4 Condensed Matter Theory Group, Department of Physics and Astronomy, Uppsala University, Box 516, S-75120 Uppsala, Sweden

**Keywords:** Cancer therapy, Smart drug delivery, Functional nanocarriers, Stimulus-responsive drug release

## Abstract

Presently, nanocarriers (NCs) have gained huge attention for their structural ability, good biocompatibility, and biodegradability. The development of effective NCs with stimuli-responsive properties has acquired a huge interest among scientists. When developing drug delivery NCs, the fundamental goal is to tackle the delivery-related problems associated with standard chemotherapy and to carry medicines to the intended sites of action while avoiding undesirable side effects. These nanocarriers were able of delivering drugs to tumors through regulating their pH, temperature, enzyme responsiveness. With the use of nanocarriers, chemotherapeutic drugs could be supplied to tumors more accurately that can equally encapsulate and deliver them. Material carriers for chemotherapeutic medicines are discussed in this review keeping in viewpoint of the structural properties and targeting methods that make these carriers more therapeutically effective, in addition to metabolic pathways triggered by drug-loaded NCs. Largely, the development of NCs countering to endogenous and exogenous stimuli in tumor regions and understanding of mechanisms would encourage the progress for tumor therapy and precision diagnosis in future.

**Graphical Abstract:**

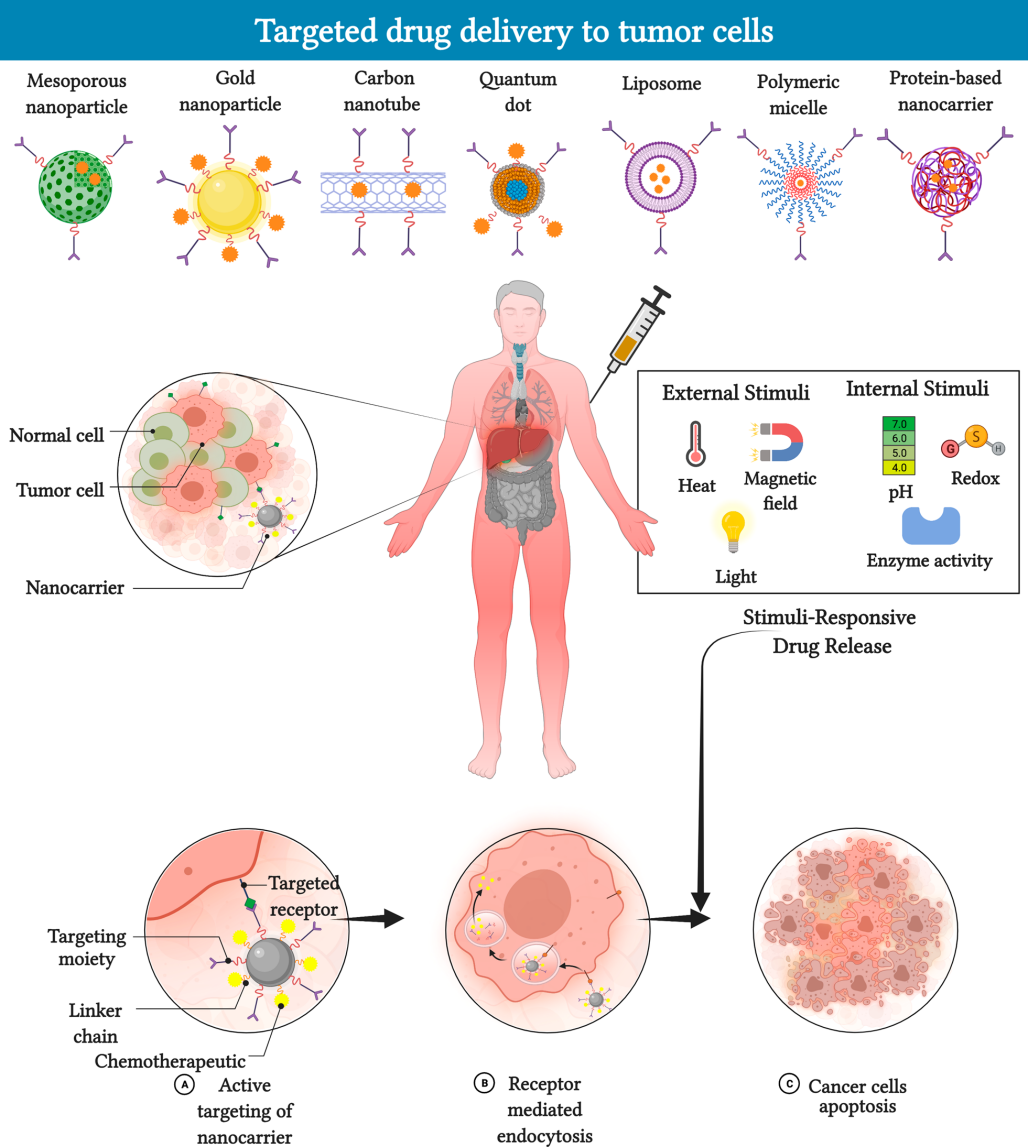

## Where are we today with cancer therapeutics?

Cancers are a group of diseases that are caused by the uncontrolled development of malignant cells, which can infiltrate tissues and spread to other regions of the body. According to the World Health Organization, cancer-related fatalities accounted for nearly 10 million deaths in 2020, and the incidence of cancer is expected to increase rapidly to 28.4 million new cases in 2040 [1]. The most common cancer-related death includes breast, lung, colon and rectum, prostate, skin (non-melanoma), and stomach cancers. Fortunately, the death rate has been reduced drastically by advances in our understanding of tumor biology and the development of improved diagnostic equipment and therapies.

### Current oncological treatments and new therapies: an overview

Several core strategies exist for treating cancer, including surgical intervention, chemotherapy, and radiation therapy, as well as a combination of these techniques. Conventional chemotherapy works primarily by interfering with the genetic material and cell division of cancer cells; however, this approach is non-selective and damages even the healthy cells, thereby resulting in severe side effects and a high mortality rate. In addition, hydrophobic drugs have poor accessibility that reduces the final drug dosage delivered to the tumor tissues, meaning that higher doses must be administered systematically. However, this can lead to severe toxicity in normal tissues and increase the chances of multiple drug resistance (MDR) where cancer cells can evade chemotherapies by developing resistance against cytotoxic drugs immediately after therapy. Therefore, novel drug delivery systems that can enhance specific targeting and reduce adverse side effects in cancer tissues are urgently required. These shortcomings of conventional chemotherapy have prompted the development of smart monitored nanocarrier (NCs)-based drug delivery systems that allow targeted drug release at specific sites and reduce toxicity [2–5] with enhanced penetration [6].

The correlation between drug delivery and nanoparticles (NPs) was first described by Paul Ehrlich [7] using the magic bullet concept, while Speiser et al. [8] were the first to report the regulated sustained release of drugs using a bead polymerization technique. Also, some engineered bioinspired synthetic and cellular systems towards design of nanomedicine platforms for the treatment of cancer [9]. In recent years, an increasing number of studies have investigated tumor biology and reported the construction of NCs using versatile materials, such as inorganic carriers, lipids [10], proteins [11], and polymeric micelles [12, 13]. This has led to the development of NC-based drug delivery systems that can deliver chemotherapeutics into the tumor microenvironment on demand. Compared to conventional chemotherapeutics, NCs like liposomes, micelles, and nanoparticles have a variety of advantageous features for use in clinical cancer therapy. For instance, NCs can have a high selective accumulation rate in the tumor microenvironment via the enhanced permeability and retention (EPR) effect [14], which improves treatment efficiency by reducing toxicity in normal tissues. Moreover, active targeted delivery can be achieved using NCs loaded with chemotherapeutic agents and conjugated to molecules that bind to receptors that are overexpressed on cancer cells.

In this review, we highlight various NCs-based drug delivery systems and discuss the targeted mechanisms via which they improve the therapeutic index of chemotherapeutic drugs. In addition, we discuss several endogenous and exogenous stimuli-responsive drug release studies in the context of present-day NCs development, in addition, the metabolic pathways and mechanisms induced by drug-loaded NCs.

## NCs used for drug delivery in cancer therapy

Several innovative drug delivery strategies are currently being used to treat cancer, and novel cancer therapies have been developed using an array of nanomaterials, including organic and inorganic particles, and synthetically produced lipids, proteins, and polymers. The delivery of drugs encapsulated in NCs offers several advantages over the direct administration of refined chemotherapeutic drugs. These include enhanced drug delivery, protection of the encapsulated drugs against degradation in the bloodstream, targeted drug delivery, efficient treatment with reduced systemic toxicity, improved drug solubility, and enhanced pharmacodynamic and pharmacokinetic drug properties [13, 15–18].

**Fig. 1:**
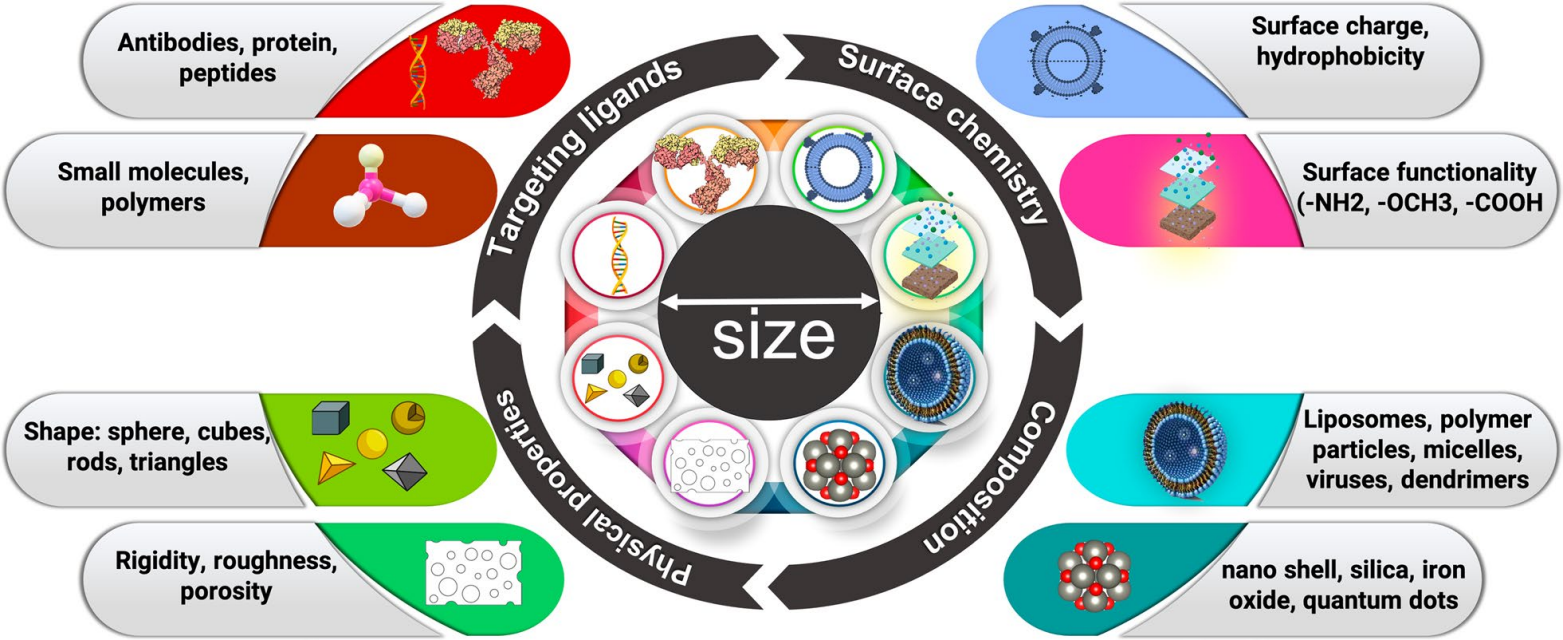
Intracellular applications of nanocarriers designed using different materials. The type and functionality of the nanocarrier are controlled by its shape, size, and targeting ligands, leading to high maneuverability and target-specific drug delivery

NPs have a small diameter of 1–100 nm and a high surface area to volume ratio, which significantly affects their biological activities and allows them to bind, absorb, and transport drugs, DNA, RNA, proteins, and detection molecules. The therapeutic efficacy of NCs are based on the efficiency of nano-drug delivery used in medical treatments which are controlled predominantly by their size, shape, and surface [19]. To date, a remarkable variety of drug delivery NCs have been developed with different sizes, molecules, conformations, and surface physicochemical characteristics (Fig. 1). It is noteworthy that conventional NCs are unspecific, unstable, shows reduced biocompatibility, low permeability, retention effect, drug resistance, and high toxicity due to several barriers encountered during circulation or abnormal vascular networks in tumor microenvironment [20]. However, the targeted stimuli-responsive NCs are precise, shows high stability, biocompatibility, enhanced permeability, shows reduced toxicity and retention effect i.e., exhibit smart behaviors at biological environments. Moreover, in conventional NCs the drug delivery is not targeted and the doesn’t show enhanced permeability and retention (EPR) effect to the tumor site, in contrast, delivery of drugs encapsulated in targeted stimuli responsive NCs offers several advantages over the direct administration of refined chemotherapeutic drugs and reduction of side effects that improves therapeutic efficacy [13, 15, 16].

Here, firstly we discuss the main types of NCs that have been used as drug delivery systems in the treatment of cancer (Tables 1 and 2).

**Table 1:** Inorganic drug delivery NCs in cancer therapy

Material	Description of carrier	Material advantage	Specificity	Refs.
CNT	Anti-P-glycoprotein antibody functionalized CNT-doxorubicin	Defeats multidrug resistance	Leukemia cells	[31]
CNT	Multi-walled CNT decorated with guanidinylated dendritic molecular transporters	Efficient DOX delivery	Prostate cancer cells	[32]
CNT	PEG-CNT complex	Mitochondrial targeting	Lung cancer cells	[33]
Layered double hydroxide NPs	Co-delivery of 5-fluorouracil and siRNAs	Prevents drug resistance and enhances cancer treatment	Various cancer cells	[34]
Layered double hydroxide NPs	Raloxifene intercalated into the interlayer gallery of LDH host	Improves therapeutic efficacy, reduction of adverse side effects	Solid tumors	[35]
Iron oxide NPs	Phospholipid-PEG-coated superparamagnetic iron oxide NPs	Chemotherapy and hyperthermia treatment	Solid cancers	[36]
Magnetic NPs	Pluronic F127-anchored iron oxide NPs	Active and passive delivery of hydrophobic drugs	Folate-positive cancer cells	[37]
Magnetic NPs	Chitosan-coated superparamagnetic iron oxide NPs	Doxorubicin delivery	Ovarian cancer cells	[38]
Mesoporous silica NPs	Azobenzene-modified mesoporous silica for NIR-triggered anticancer drug delivery	Drug release rate can be controlled by varying the intensity and/or time	Solid tumor	[39]
Mesoporous silica NPs	Hyaluronic acid-capped mesoporous Silica NPs	Site-selective, controlled-release delivery	MDA-MB-231 and NIH3T3 cells	[40]
QDs	Riboflavin-tageting graphene quantum dots-PEG-benzofuran	High potency, improved dispersibility	Laryngeal, lung and colorectal cancer cells	[2]
QDs	Hyaluronic acid/ferrocene (Fc)-anchored nitrogen-doped graphene QDs (Fc-GQD-HA)	Selective binding to CD44 receptors, redox-based drug delivery	Diverse range of cancer cells	[41]
QDs	Hederagenin anchored black phosphorus QDs encapsulated with platelet membrane	Mono-dispersive capacity, elevated drug-loading	In vivo application	[42]

*CNT* carbon nanotubes; *NPs* nanoparticles; *QDs* quantum dots; *LDH* layered double hydroxides; *PEG* polyethylglycol

**Table 2:** Organic drug delivery NCs in cancer therapy

Material	Description of carrier	Material advantage	Specificity	Refs.
Liposomes	Liposomal doxorubicin	Improved delivery to site of disease; decrease in systemic toxicity of free drug	Ovarian cancer; multiple myeloma	[99]
Liposomes	Liposomal daunorubicin	Improved delivery to tumor site; lower systemic toxicity arising from side effects	Karposi’s sarcoma	[100]
Liposomes	Genistein and plumbagin encapsulated nanoliposomes	Inhibition of cell metabolism	In vitro and in vivo prostate cancer	[101]
Liposomes	Folate-conjugated bovine serum albumin bound paclitaxel NPs	Increased solubility, cellular uptake; targeted specificity	Prostate cancer cells	[102]
Protein-based	Alpha mangostin loaded crosslinked silk fibroin-based NPs	Physicochemically stable, increased the drug's solubility	Colorectal and breast cancer	[85]
Protein-based	Noscapine-loaded human serum albumin NPs	High drug-loading efficiency (85–96%) and delivery of maximum quantity of drug to the tumor site	Breast cancer cells	[103]
Protein-based	Plasmid cDNA (pGL3) polyethyleneimine (PEI)-coated HSA NPs	Enhance endosomal escape	In vitro gene delivery application	[104]
Micelles	Polymeric methoxy-PEG-poly(D,L-lactide) micelle formulation of paclitaxel	Improved delivery to site of disease; decrease in systemic toxicity of free drug	Breast cancer; ovarian cancer	[91]
Micelles	Folate-PEG/Hyd-curcumin/C18-g-polysuccinimide	pH sensitive drug release	Colon cancer	[105]
Micelles	PEGylated prodrug nano-micelles	Glucose-sensitive	In vitro and in vivo anticancer activity	[106]
Polymeric Micelles	CD44v6-targeted polymeric micelles (PM) loaded with niclosmide	Increase drug safety	Efficacy against colorectal stem cells	[107]
Self-assembly	Aptamer-tethered DNA assembly	Stronger targeting ability, higher cellular uptake	Cancer cell imaging	[108]
Self-assembly	DNA-aptamer conjugated RNA-triple helix hydrogel	Efficient cellular uptake and enhanced nuclease resistance with superior biocompatibility	Triple negative breast cancer detection and treatment	[109]
Self-assembly	Folate-modified MPEG-PCL	Improved bioavailability, low toxicity, sustained drug release	Colorectal cancer mice model	[110]
Self-assembly	Folate receptor-targeted β-cyclodextrin (β-CD)	Biosafety, bioavailability, and improve curcumin drug loading capacity	Cervical cancer, fibroblast cells	[111]

### Inorganic NCs

**Fig. 2:**
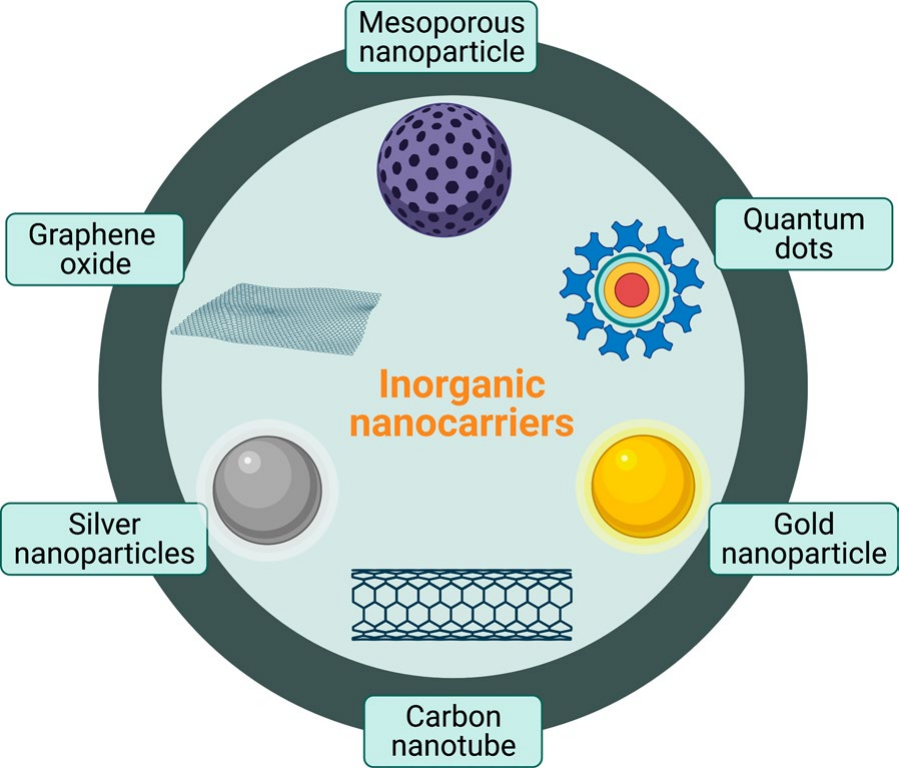
Examples of inorganic nanocarriers. Mesoporous nanoparticles are silica nanoparticles with overall diameter of < 1 μm and pores diameter from 2 to 50 nm. Quantum dots are colloidal fluorescent semiconductor nanocrystals (2–10 nm). Silver and gold nanoparticles (1–100 nm) have high surface area, tunable optical, and are non-toxic. Carbon nanotubes consist of coaxial graphite sheets (< 100 nm) rolled up cylindrical. Graphene oxide is single atomic layer of carbon, consist of thickness 1 nm. Figure has been created using Biorender

Inorganic NCs have proven to be highly useful in cancer therapy owing to their high surface area to volume ratio and easy conjugation with various cancer drugs. In addition, biocompatibility, low toxicity, and the ability to control drug release have facilitated the use of inorganic NCs, such as mesoporous silica NCs (MSNCs), gold NCs (AuNCs) [21], magnetic NCs (MNCs), carbon nanotube NCs (CNT-NCs), graphene oxide and quantum dots (QDs), as drug carriers (Fig. 2) [22].

#### Mesoporous silica NCs

MSNCs have a variety of unique characteristics that make them appealing NCs, including a highly porous structure and adsorption capacity, tunable particle size, easy functionalization, excellent biocompatibility, and the potential to act as a physiological container to protect drugs against dysfunction or denaturation [23]. In addition, the pore diameter of some MSNCs can be controlled to regulate drug encapsulation ratios and the release kinetics, allowing anticancer medicines to be delivered in a tailored manner and discharged on time to promote cellular uptake without being released before reaching the intended target site. MSNCs can also adsorb various hydrophobic drugs and thus act as ubiquitous intracellular drug carriers by transporting them across the cell membrane. Moreover, MSNCs can simultaneously adsorb both hydrophilic and hydrophobic drugs for targeted drug delivery. For instance, Chan et al. synthesized phenyl-exMSN-PEG + TA (NTT2_131), which was anchored simultaneously with hydrophilic doxorubicin (DOX) and phobic JM15 anticancer drugs [24]. Moreover, MSNCs have the ability to target the tumor site through both active and passive mechanisms, such as the EPR effect and the evasion of reticuloendothelial system (RES) clearance [25]. Furthermore, the surface of MSNCs can be functionalized with specific ligands, such as hyaluronic acid or transferrin, to target specific tumors or be coated with proteins, enzymes, and magnetic nanoparticles, which function as homing devices. Together, the intracellular absorption, pharmacokinetics, and tissue distribution patterns of MSNCs throughout the targeting process can be significantly altered by regulating the particle size and surface of MSNCs.

#### Gold nanocarriers

AuNCs have been used as anticancer agents in photothermal therapy (PTT) owing to their excellent ability to convert light into heat [26]. The therapeutic potential of AuNCs is determined by their size, shape, surface plasmon resonance (SPR), and surface biochemistry [27]. Basically, size and shape of nanoparticles are important parameter that effects cellular uptake and site-specific drug delivery from the synthesized system. It was also noticed that the smaller particles infused into the deep layers of tissues while the 100–200 nm particles stayed on the surface [28], that additionally signifies better bioavailability of small sized gold NPs. The particle size also affect the blood concentration along with circulation time. Moreover, their benign nature and excellent surface functionalization properties make AuNCs effective anticancer drug carriers. The surface functionalization of AuNCs with anticancer drugs can improve their anticancer efficacy via the combined effect of the nanoparticle–drug conjugate on tumor cells, while anchoring with a specific antibody can improve their target specificity [29]. Furthermore, the surface modification of Au NPs with a pH-sensitive material can allow them to specifically target tumor cells, which have a lower pH than normal cells. Chaudhari et al. [30] investigated the metabolic pathways and mechanisms induced by methotrexate (MTX)-AuNC in breast cancer cells. MTX-AuNC targeting the folate receptor, demonstrated considerable uptake by the breast cancer cells, as well as substantial down-regulation of the anti-apoptotic gene and up-regulation of pro-apoptotic genes [30].

#### Magnetic NCs

MNCs have recently received a lot of interest owing to their immense potential as heat regulators for treating hyperthermia as well as their ability to transport drugs in a targeted manner, which can reduce systemic effects [43]. In addition, MNC-based cancer therapies have novel features that are not observed in traditional approaches, such as minimal toxicity and controlled drug release. The behavior of MNCs is predominantly affected by their hydrodynamic diameter, surface chemistry, and magnetic properties. Surface chemistry is crucial for preventing clearance by the RES and improving half-life within the blood circulation. For example, researchers have modified the surface of MNCs with neutral and hydrophilic ligands, such as PEG or P(S/V-COOH) polymers, and reported an improvement in their circulatory half-life from minutes to months [44]. Anchoring the surfaces of MNCs with new ligands has facilitated targeted drug delivery, and the anchoring of single or multiple drugs on MNCs has recently been studied to improve anticancer drug efficacy. The stability and biocompatibility of MNCs can be optimized by anchoring them with organic or inorganic compounds, which can improve the efficacy of anti-cancer chemotherapy and gene therapy [45]. It is worth to mention that MNCs, offers a suitable tool for cancer treatment and diagnosis due to their unique features which distinguish them from other NCs. These can act through magnetic drug targeting, making them therapy magnetically responsive, therefore it can be controlled inside the human body by external magnets, and eventually absorbed into tumor tissues. Their utilization in magnetic resonance imaging (MRI) delivers a high divergence for generating the most detailed imaging. Such as, magnetic iron oxide NPs widely applied for lung MRI due to their good magnetization and biocompatibility as well as their appropriate drug uptake and subsequent release [46]. Similarly, water-dispersible PEI-conjugated iron oxide NPs have been also exploited for MRI-based cancer imaging [47].

#### Carbon nanotube NCs

Carbon nanotubes (CNTs) are long cylindrical structures with flexible NCs characteristics that have sparked interest as drug delivery molecules owing to their unique biological, physical, and chemical capabilities [48]. Indeed, CNTs have been used to deliver anticancer drugs, such as DOX, paclitaxel, methotrexate, and small interfering RNAs (siRNAs), to treat a range of malignancies.

The developing siRNA technology merged with chemotherapy has presented significant rationale in cancer therapies. The main challenges in the fabrication of siRNA/chemotherapeutic drug co-loaded NPs are specific targeted delivery, sufficient cargo protection, and site-specific release [49]. CNTs are a popular multifunctional drug delivery system owing to their large surface area, high adsorption capacity, durability, ease of functionalization, an exceptionally high length-to-diameter ratio, and excellent intracellular absorption [50]. In addition, the surface characteristics of CNTs can be readily altered using covalent/noncovalent crosslinking, making them a favorable nanomaterial. The hydrophobicity of CNTs leads to π–π stacking interactions with a variety of drugs and medicinal compounds, including those with an aromatic ring such as anthracyclines; however, CNTs have a low solubility that makes them ineffective drug delivery molecules. The solubility of CNTs can be improved by surface functionalization with various molecules using covalent/noncovalent bonding and electrostatic forces to make them more hydrophilic, thus altering their biocompatibility profile [51, 52].

#### Quantum dots NCs (0-D)

Quantum dots (QDs) s are uniform spherical (0-D) NCs with very small sizes of 1–10 nm [53]. These fluorescent particles have exceptional physio-chemical characteristics, including a very large surface area, biocompatibility, highly customizable photoluminescence, strong signal brightness, and high photostability. These features have inspired researchers to use QDs as potential NCs for targeted and traceable drug delivery systems, to monitor intracellular processes in real time, and for in vivo molecular imaging. However, the hydrophobicity, easy agglomeration, and high adsorption affinity of QDs for the surrounding impurities make them poor candidates for therapeutic applications. Fortunately, these shortcomings can be avoided by coating QDs with ionic species or layering them with ligand shells [54]. For instance, imaging probes, smaller hydrophobic or hydrophilic drugs, and targeting agents can be embedded between the inorganic core and the amphiphilic polymer coating layer [55, 56].

#### 2D-materials NCs

2D materials have excessive light photodynamic and heat conversion proficiency, therefore they retain various benefits in biomedical applications. These characteristics provide them high potentials in medicine fields, including imaging [57], sensing [58] and therapy [59, 60]. In the meantime, 2D materials exhibited a challenging capability in drug delivery with various advantages. One of the exclusive crucial features of 2D materials is the lamella organization, that offers the huge surface space for high drug loading efficiency [61]. Yu et al. synthesized a reduced GO nanocomposite modified with a polydopamine (PDA). They coated the dopamine-modified rGO surface with antiarrhythmic peptide 10 which limit tumor development about more than 95% while used with radiotherapy [62]. This innovative drugs possesses several abilities, such as large surface area, excellent biocompatibility, and a high drug loading capacity. Moreover, these NCs confirmed subsequent pH responsiveness and drug release. As stated by Xing et al. injectable hydrogel made of black phosphorous nanosheets and cellulose exhibits noteworthy antitumor activity in contrast to PTT. Interestingly, these nanoscale hydrogel platform is non-toxic and 100% biocompatible as confirmed by both in vitro and in vivo studies [63].

### Organic nanocarriers

Organic NCs have been studied for decades and contain a diverse range of components. They are biocompatible, have low drug loading capacity and the drug molecules are encapsulated or conjugated.

#### Liposomes NCs

**Fig. 3:**
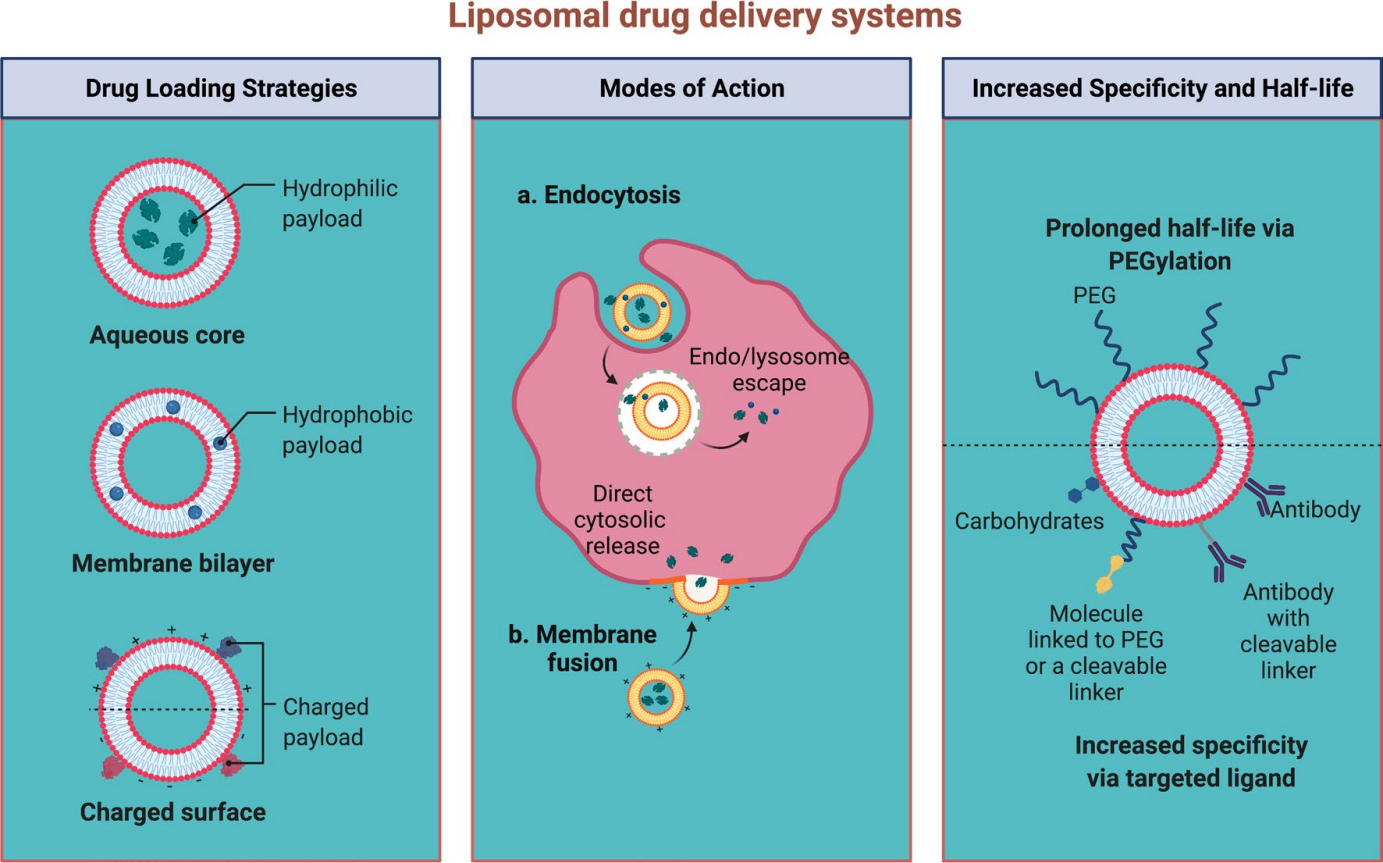
Schematic representation of the different types of liposomal drug delivery systems. **A** Conventional liposomes consist of a lipid bilayer surrounding aqueous compartments, composed of phospholipids and cholesterol unmodified **B** PEGylated liposomes have a hydrophilic polymer coating (PEG) on the surface of the liposome that modifies in vivo characteristics and behavior via steric stabilization. **C** Ligand-targeted liposomes can affect specific targets via ligands attached to the surface or terminal end of the attached PEG chains. **D** Theranostic liposomes are a single system consisting of a nanoparticle, a targeting element, an imaging component, and a therapeutic component. Figure has been created using Biorender

Liposomes are made up of an exterior lipid layer and a core that contains either hydrophobic or hydrophilic medications; they were the first nano-scale drugs to be licensed for clinical use [64]. Liposomes can be modified to perform a variety of tasks by altering the lipid layer structure; for instance, the lipid layer can be altered to mimic the biophysical properties of living cells [65], which can improve the efficiency of targeted drug delivery [66]. The inner aqueous core of liposomes can be loaded with amphiphilic drugs using various techniques, such as the ammonium sulfate gradient method [67]. However, standard liposomes are rapidly eliminated from the bloodstream and can be detected by opsonin proteins, leading to phagocytosis and destruction by macrophages. To overcome this issue, surface-modified liposomes have been developed that can be grafted with monoclonal antibodies, glycoproteins, carbohydrates, vitamins, antibodies, and peptides to actively target cancer cells (Fig. 3).

Similarly, Najlah et al. developed stealth liposomes by modifying the surface coating of a hydrophilic lipid polymer derivative of PEG [68]. This modification was shown to improve the efficacy of the encapsulated drugs by prolonging their circulation in the blood and reducing their elimination [69].Unfortunately, the reliability of the PEGylated liposomes decreases upon systemic injection, which may prolong their circulation in the blood; therefore, novel PEG-dendron phospholipids have been developed to produce super-stealth liposomes. In vitro studies have shown that ligand-targeted liposomes can promote the internalization of liposome-drug conjugates via prostate-specific membrane antigens on the targeted cell [10]. Decades of liposome research have facilitated the development of liposomes that are a suitable platform for the in vivo administration of various anti-cancer drugs, including docetaxel [70], and nucleic acids [71]. Indeed, liposomes are now increasingly being used to treat breast [72] and prostate [73, 74] cancers. In addition, more liposome-based medicines are currently in clinical trials for cancer therapy [75, 76].

#### Protein-based NCs

**Fig. 4:**
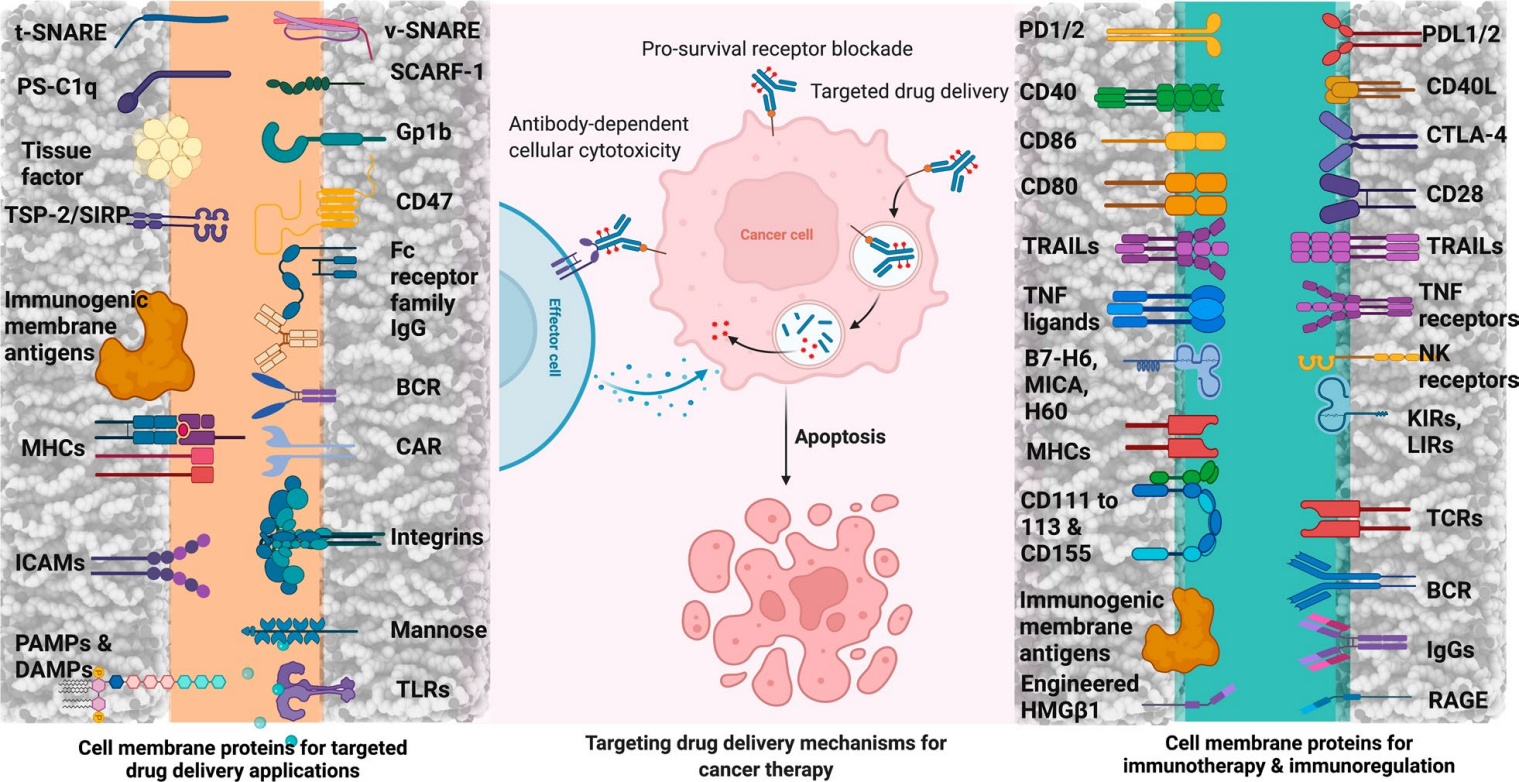
Potential cell surface proteins and their complementary receptors for use in targeted-drug delivery applications. *t-SNARE/v-SNARE* target snap receptor/vesicle snap receptor; *PS* phosphatidylserine; *C1q* complement component 1q; *SCARF-1* scavenger receptor class-F, member-1; *Gp1b* glycoprotein-Ib; *TSP-2* thrombospondin-2; *SIRPα* signal regulatory protein α; *CD* cluster of differentiation; *ICAM* intercellular adhesion molecule; *LFA-1* lymphocyte function-associated antigen-1; *MAC-1* macrophage adhesion ligand-1; *VLA* very late antigen; *PAMP* pathogen associated molecular pattern; *DAMP* damage-associated molecular pattern; *PD-1/PD-2* programmed cell death protein-1/programmed cell death protein-2; *PD-L1/PD-L2* programmed death-ligand-1/programmed death-ligand-2; *CTLA-4* cytotoxic t-lymphocyte-associated protein-4; *TRAIL* tumor necrosis factor-related apoptosis-inducing ligand; *TNF* tumor necrosis factor; *B7-H6* B7 homolog 6; *MIC* MHC class I polypeptide-related sequence; *H60* histocompatibility protein-60; *NKp* natural cytotoxicity triggering receptor; *NKG* natural killer cell granule protein; *KIR* killer-cell immunoglobulin-like receptor; *LIR* leukocyte immunoglobulin-like receptor; *HMGβ1* high-mobility group protein β1; *RAGE* receptor for advanced glycation end products. Figure has been created using Biorender

Proteins are essential macromolecules that possess unique functions and characteristics in biological materials and manufacturing field; therefore, they are utilized as a starting material for the synthesis of NCs (Fig. 4) [77, 78].

Since protein-based NCs display good biodegradability and low toxicity, they are used to carry both pharmaceuticals and nutraceuticals [79]. Additionally, protein NCs have high stability, are non-antigenic, can be metabolized, and it is easy to synthesize their particles, modify their surface, and monitor their size [11]. Owing to their promising characteristics, protein NCs have been used for a variety of targeted uses, such as cancer therapy [80, 81], pulmonary delivery [82], and vaccine delivery [83]. Protein NCs can be incorporated into biodegradable polymers in a monolithic particle structure, such as a microsphere [84], to allow controlled and sustained drug release and can be produced using proteinaceous materials such as silk protein fibroin [85], bovine [86], human serum albumin [87], and gliadin [88].

#### Micelles NCs

**Fig. 5:**
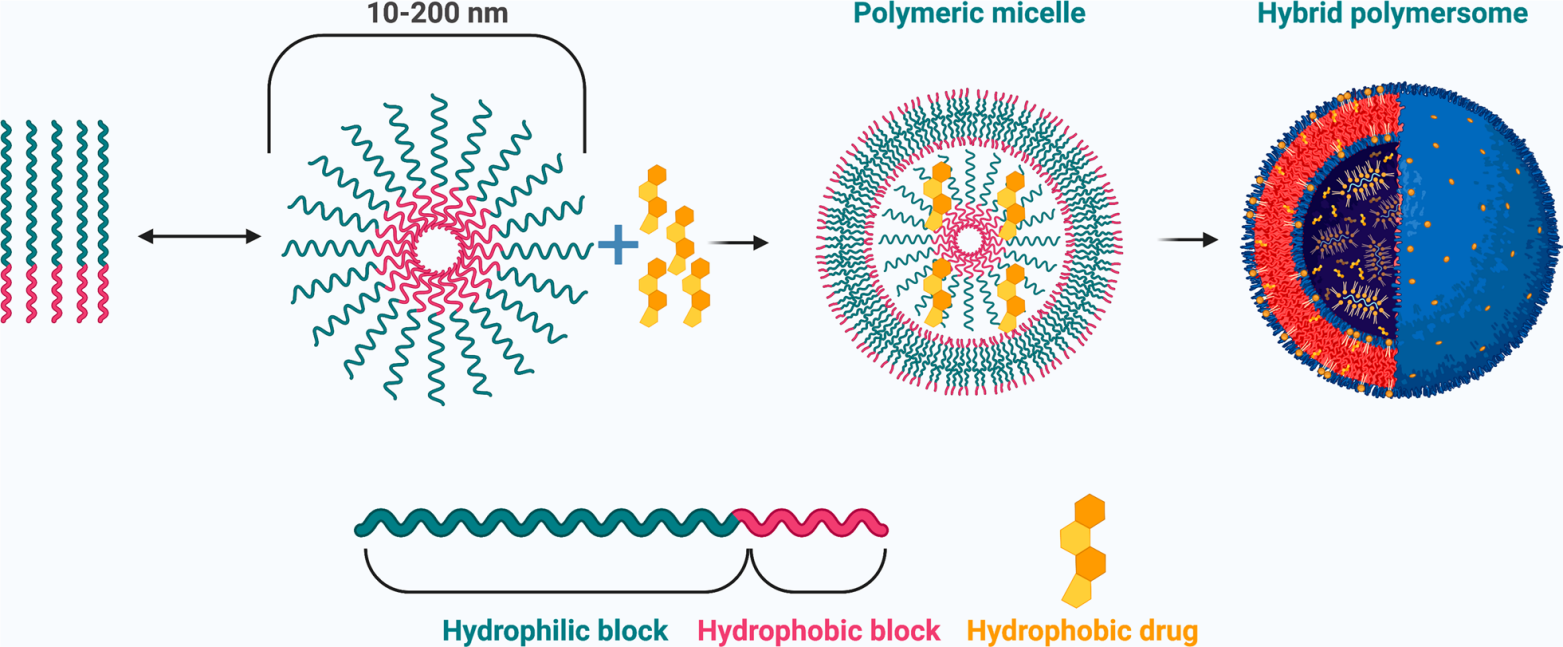
Schematic representation of polymeric micelles. Self-assembly of di-block copolymers into a polymeric micelle takes place above the critical aggregation concentration. The hydrophobic drug is encapsulated into the hydrophobic core. Figure has been created using Biorender

Micelles are spherical nanosized structures that are formed via the self-assembly of amphiphilic block copolymers that have both hydrophilic and hydrophobic portions in aqueous solution, thus forming a hydrophobic core and a hydrophilic shell, known as polymeric micelle (Fig. 5) [89, 90]. Micelles can be formed intrinsically at specific concentrations (critical micelle concentration (CMC) and temperatures. For instance, if the solvent portion is hydrophilic and its concentration exceeds the CMC, the polar region of the co-polymer is drawn toward the solvent, whereas the hydrophobic region is repelled from the solvent [12]. In polymeric micelles, the hydrophobic core serves as a reservoir site for the incorporation of hydrophobic drugs, while the hydrophilic shell stabilizes the core and keeps the polymer and drug intact, making micelles a suitable candidate for intravenous administration. Genexol-PM [PEG-poly(D,L-lactide)-paclitaxel] was the first cremophor-free polymeric micelle to contain paclitaxel and was shown to have no adverse reactions and a good toxicity profile in advanced refractory cancers [91].

#### Self-assembled drug NCs 

The spontaneous organization of molecules into ordered geometric structures is known as molecular self-assembly and provides both inorganic and organic structures with unique qualities via non-covalent interactions. The spontaneity, versatility, inexpensiveness, and simplicity of self-assembly mean that it is extensively used for designing nanoscale biomaterials [92] and has various biomedical applications, including drug delivery, tissue engineering, and regenerative medicine. Self-assembled NCs such as micelles, polymeric NPs, liposomes, and CNTs have been shown to improve drug delivery, prolong blood circulation, control drug release kinetics, impart molecular targeting, improve tumor accumulation [93] and bioavailability, allow proper encapsulation, and protect the drug from the external milieu [94]. Ultimately, these self-assembled NCs help to overcome physiological barriers in vivo during drug delivery.

#### Supramolecules

Supramolecules are molecular assemblies that are held together by electrostatic interactions, metal coordination, hydrophobic attractions, host–guest interactions, and van der Waals forces [95]. Also, supramolecular compounds are easy to realize the “Lego-like” construction of NCs, which have excellent editability. These interactions provide the NCs with stability in body fluids and improve their characteristics, including durability, sustained release, and transport efficiency. Accordingly, supramolecules are used as drug carriers for targeted anticancer delivery systems [96]. Indeed, the side effects of anticancer drugs, such as DOX, on normal cells can be controlled by using amphiphiles to generate supramolecular aggregates for cancer therapy [97].DOX-anchored supramolecular polymersomes circulate in the bloodstream for a longer time, and in vivo experiments have demonstrated their superior antitumor efficacy against malignant HeLa cells with decreased cytotoxicity [98].

## Stimuli-responsive NCs drug release

**Fig. 6:**
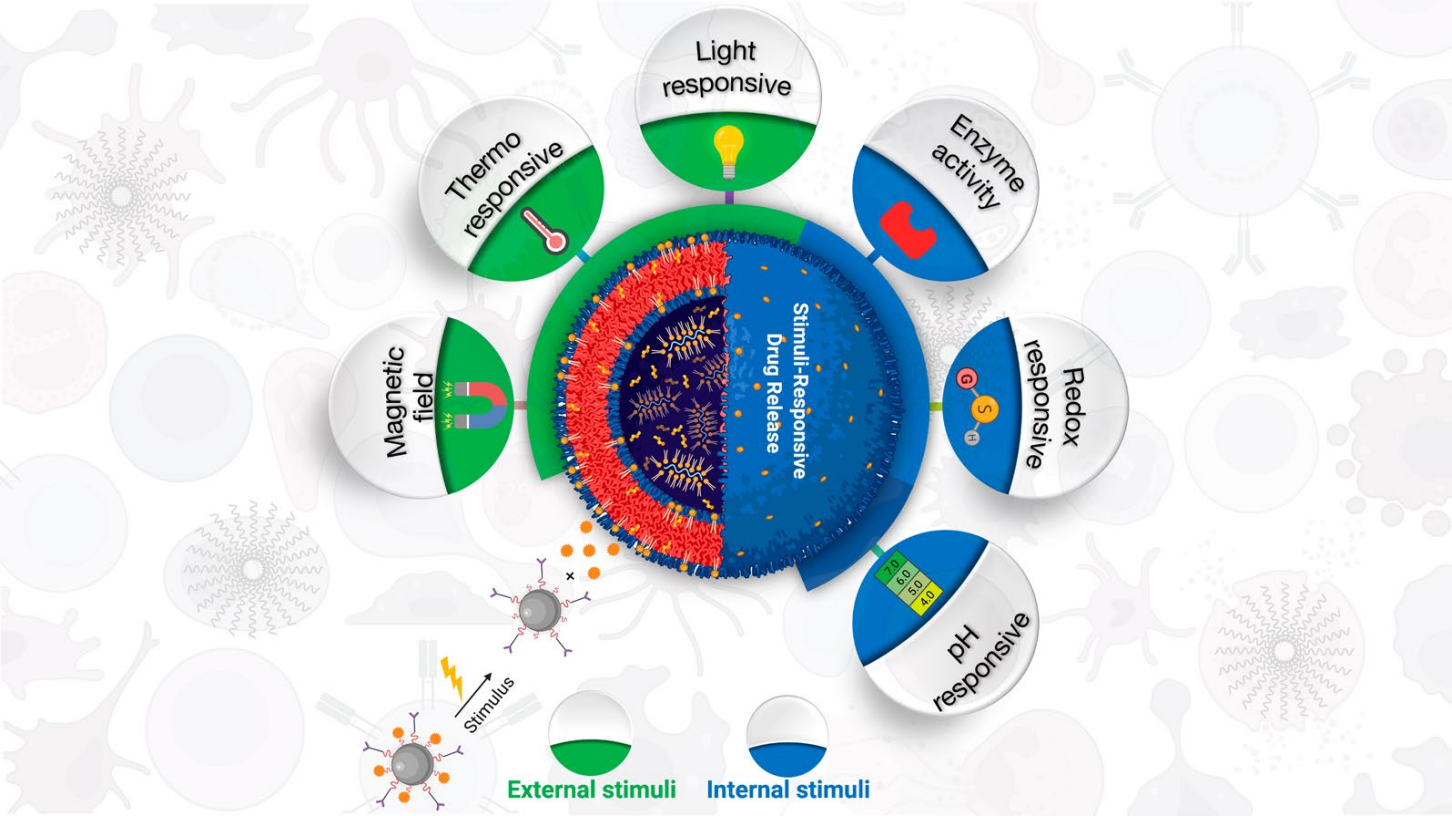
Schematic illustration of drug release. In response to either internal (pH, redox, enzyme) or external (thermo, magnetic field, light) stimuli. Figure has been created using Biorender

In recent decades, the development of novel polymers has prompted the study of smart stimuli-responsive drug delivery systems [112, 113]. Wei et al. proposed that stimuli-responsive nanomedicines determine noteworthy benefits via their adaptive transition application during drug delivery cascade [114]. A number of literature suggests that stimuli-responsive materials improve their pre-designed functions in response to the tumor microenvironment (TME) or intracellular signals, for instance conversion of the surface charge, PEG deshielding, exposure of tumor-targeting ligand, and controlled drug release [115–117]. It is broadly known that the TME has distinctive physiological characteristics such as acidic pH [118], hypoxia [119], and induction of certain enzymes [120]. Overall, the NCs might response to exogenous stimuli, including temperature, magnetic field, light etc., and endogenous stimuli, including pH, ATP, H_2_O_2_, enzyme, redox-potential, and hypoxia etc., although the stimuli might be emerged in TME or inside cancer cells (Fig. 6) [121].

### Endogenous stimuli

Also known as an intrinsic stimuli, endogenous stimuli include changes in the internal pH levels, enzyme activity, redox activity, hypoxic conditions of the body.

#### pH-responsive NCs

Tumor cells primarily generate energy by enhancing glycolysis and then fermenting lactic acid in the cytosol, which is known as the Warburg effect [122]. The increased acid production results in an acidic pH in and around the cancer cells compared to that in normal cells, which is why pH has been established as an effective physiological property for targeted and controllable drug delivery by pH-responsive NCs [123, 124]. To achieve targeted and site-specific drug delivery, pH-responsive NCs exploit pH gradients in the physiological milieu [125]. The normal physiological pH is neutral (7.0–7.4), whereas the pH of the tumor microenvironment is highly acidic due to high metabolic activity and inadequate perfusion [126]. The main feature of pH-triggered NCs is their transition from hydrophobic to hydrophilic in response to acidic pH levels, which can alter their size or behavior. For instance, Tang et al. reported pH-sensitive NC systems based on reversibly ionizable carboxylic groups that undergo protonation and deprotonation due to changes in pH, leading to NC swelling, rupture, and drug release [127].

Several pH-responsive materials have been used for controlled drug delivery, including curcumin-loaded pH-sensitive N-naphthyl-N,O-succinyl chitosan polymeric micelles that were developed for colon-targeted drug delivery and exhibited pH-dependent release kinetics in colorectal cancer cells [128]. In addition, Ishida et al. [69] synthesized various pH-sensitive sterically-stabilized liposomes composed of DOPE/HSPC/CHEMS/CHOL/mPEG_2000-_DSPE. These liposomes enhanced drug retention and pH-sensitivity, increased the blood retention time of encapsulated DOX, and targeted antigens on cancer cells to allow controlled drug release in the acidic tumor microenvironment [69]. Unfortunately, tumor cells can still become resistant to anticancer drugs delivered by NCs, thus compromising their therapeutic efficacy. To address this issue, Guo et al. [129] developed a novel dual pH-sensitive micelle NC, PEO-b-P(DMAEMA-co-MAEBA) combined with a specific inhibitor (A01) of the TMEM16A ion channel, which is overexpressed in lung adenocarcinoma tissue. These micelles released A01 into the surrounding weakly acidic microenvironment of lung adenocarcinoma cells but were also internalized by the cells and entered their endosome-lysosome compartments, where they inhibited TMEM16A and MAPK signaling. pH-sensitive self-assembled drug carriers have also been extensively used. For instance, Song et al. synthesized biodegradable pH-responsive PEGylated DOX micelles that self-assemble in aqueous solutions via esterification and Schiff base reactions [130]. These NCs had a high drug payload and an excellent pH-responsive controlled drug release profile, as well as rapid prodrug internalization and enhanced antitumor activity against MCF-7 cancer cells. Interestingly, Chen et al. prepared a nature-inspired pH-responsive organic–inorganic hybrid capped mesoporous silica NPs based on biomineralization mechanism having low toxicity, enhanced cellular uptake and efficient drug release [131]. In another study, a group of researchers established a cRGD-decorated pH-responsive polyion complex micelle for DOX intracellular targeted delivery to enhance tumor inhibition and decrease cytotoxicity [132].

#### Redox-responsive NCs

Glutathione sulfhydryl (GSH) is a tripeptide compound in which glutamic acid is conjugated through its side chain to the N-terminus of cysteinylglycine. Since GSH levels are fourfold higher in cancer cells than in normal cells [133], it is used to produce redox-responsive NCs. In particular, GSH cleaves the disulfide bond of NCs in cancer cells, leading to controlled redox-driven drug release [134].

Various redox-responsive drug delivery systems have been reported in recent years, including liposome, micelle, nanogel, and prodrug-based systems. Redox-responsive liposomes initiate drug release in the presence of GSH (reducing agents) via disulfide linker reduction or liposome membrane destabilization. For instance, Chi et al. [135] synthesized a novel Chol-SS-mPEG/HA liposome (conjugated with PEG via disulfide linkages) that displayed cytoplasmic drug release triggered by GSH due to PEG de-protection and liposome agglomeration. In another study, Yin et al. [136] prepared chitooligosaccharides (COS) conjugated with cholesterol through a disulfide linker, wherein linker reduction caused COS removal, liposome instability, and rapid drug release. Micelle-based drug delivery systems are often used because of their ease of administration, enhanced circulation time when PEGylated, high structural stability, good hydrophobic drug encapsulation, and ability to respond to the external environment [137]. Micelles assembled using disulfide linkages are redox-responsive, as they can be cleaved in the presence of GSH (or a reductive environment). Sun et al. prepared redox-responsive amphiphilic glycan conjugates by linking heparosan with deoxycholic acid via disulfide bonds [138]. When the micelles reached cancer cell cytosol, they rapidly disassembled and released the drug. Similarly, Kang et al. [139] developed a redox-responsive system by linking gambogic acid with poly(amido amine)s via amide bonds, which self-assembled into micelles. The presence of disulfide bonds in a reductive environment led to rapid disassembly and release of the encapsulated drugs.

Although disulfide bonds are the main component when designing redox-responsive NCs, diselenide bonds can be used to prepare mesoporous silica-based NPs [140]. For instance, controlled drug release can be achieved using albumin or myoglobin by conjugating them to mesoporous silica-based NPs via diselenide bonds, which yields an NC that is highly responsive to GSH and H_2_O_2_ [141]. In addition, Lei et al. developed a novel intrinsic redox-responsive metal–organic framework carrier using iron, aluminum, or zirconium as metal nodes and 4,4-dithiobisbenzoic acid (4,4-DTBA) as the organic ligand [142].

#### Enzyme-responsive NCs

Upregulation of several enzymes are linked to the pathophysiology of many diseases, including infection, inflammation, and cancer. Since most of the enzymes are present in similar concentrations in both cancerous and normal cells [143], these NCs cannot be used for intracellular drug release; however, enzyme-cleavable peptides can be used to generate enzyme-triggered NC deshielding, which can ultimately allow the drug to be released. Enzyme-triggered NCs can be prepared by modifying the NC surface, which can respond to the biocatalytic reaction of enzymes that are overexpressed in the extracellular microenvironment of cancer cells. Such as, matrix metalloproteinases (MMPs) and hyaluronidases (HAs) are the extracellular enzymes that are mostly upregulated in tumors [144, 145]. The upregulation of these extracellular enzymes can be used to trigger NCs for reduction of their size and surface ligand exposure for tumor infiltration. It is well-stated that cathepsin and legumain showed overexpression in several cancer cells [157–161]. Legumain is the endopeptidase which specifically cleaves linkers containing asparagine or aspartic acid residues which are upregulated in the various cancer cells lysosomal compartments. For instance, Liu et al. [162] developed a legumain cleavable liposome consisting of alanine-alanine-asparagine substrate linked to cell penetrating peptides (trans-activating factor) carrying doxorubicin. In another study, Cai et al. [146] developed an enzyme-responsive colon-specific delivery system based on hollow mesoporous silica spheres conjugated with biodegradable chitosan via cleavable azo bonds and encapsulating DOX. This NCs system displayed enzyme-responsive drug release in the presence of colonic enzymes.

#### Hypoxia-responsive NCs

In solid tumors, the poor vascularization system is possibly to form low oxygen level (hypoxia), that plays a critical role in cancer progression, including distant metastasis [147]. Consequently, many approaches have been employed for treatment of hypoxic tumors, primarily including rising the oxygen level and by means of hypoxia activatable drugs, etc. [119]. Till date, different types of nanocarriers have been designed for the drug delivery system to hypoxic tumors [148], such as silica nanoparticles [149], liposomes [150], layer-by-layer nanoparticles [151], polymeric micelles [152], nanovesicles [153], polymersomes [154], and albumin nanoparticles [155] (Table 3).

**Table 3:** List of representative hypoxia-responsive NCs [112]

Nanocarriers	Magnetic-responsive strategy/materials	Cargos	Applications
Liposomes	The prodrug of banoxantrone dihydrochloride (AQ4N) could be activated in hypoxic environment caused by PDT	Ce6, AQ4N	Cancer therapy
Silica nanoquencher	Azo monomer; cell-penetrating poly(disulfide)s (CPD) coated silica nanoquencher (BS-*q*NP) (CPD-protein@BS-*q*NP)	Antibody (Cetuximab), fluorescent dye	Hypoxia-triggered protein release and fluorescence imaging
Upconversion nanoparticles (UCNPs)	Oxygen indicator [Ru(dpp)_3_]^2+^Cl_2_ for hypoxia detection as UCNPs provided the excitation light of [Ru(dpp)_3_]^2+^Cl_2_ by upconversion process at 980 nm	[Ru(dpp)_3_]^2+^Cl_2_, UCNPs	Imaging hypoxic regions or oxygen changes in cells and zebrafish
Nanoparticles	The photosensitizer of ICG-mediated PTT induced hypoxia, which then activated the prodrug of TPZ	TPZ, ICG	Tumor therapy by PDT and chemotherapy
Nanoparticles	The shift from hydrophobic to hydrophilic of 2-nitroimidazole that grafted to polymers in light-activated hypoxia	Doxorubicin, light-sensitive polymer	Hypoxia-triggered drug release, tumor
Nanoparticles	PEG-azo(azobenzene)-PEI-DOPE block copolymer	siRNA	siRNA delivery and tumor RNAi
Nanoparticles	Layer-by-layer nanoparticles with a pH-sensitive layer for targeting of tumor hypoxia	Sulfonated polystyrene beads or carboxylated quantum dots	Systemic tumor targeting
Cancer cell membrane coated MOFs	The porphyrinic MOFs could generate toxic ROS for PDT and cause hypoxic regions for activating TPZ	Porphyrinic metal organic framework, TPZ	Tumor targeted PDT and chemotherapy
Nanovesicles	The light irradiation of Ce6 induced hypoxia for oxidation bioreduction of 2-nitroimidazole in polymers and activation of TPZ	Ce6, TPZ	Tumor fluorescence imaging and therapy
Polymeric micelles	The metronidazole (MN) grafted in polymers could change hydrophobicity in hypoxic conditions for drug release	Doxorubicin	Tumor chemotherapy and radiotherapy
Polymersomes	The PLA (polylactic acid)-azobenzene-PEG is sensitive to hypoxia	Gemcitabine, hypoxia-sensitive dye “Image-iT”	Tumor imaging and drug delivery
Albumin nanoparticles	With hypoxia-sensitive azobenzene linker to covalently bridge photosensitizer Ce6-conjugated HSA and oxaliplatin prodrug-conjugated HSA	Oxaliplatin prodrug, Ce6	Tumor chemotherapy and photodynamic therapy
Mesoporous silica nanoparticles	The Ce6-dopped mesoporous silica nanoparticles were decorated with PEG and glycol chitosan by hypoxia-sensitive azobenzene linker	Oligonucleotide (CpG), Ce6	Cancer immunotherapy
Solid-state sensors	Iodide-substituted difluoroboron dibenzoylmethane-poly(lactic acid) [BF_2_dbm(I)PLA] solid-state sensor material	BF_2_dbm(I)PLA	Tumor hypoxia optical imaging
Polymeric probes	Poly(N-vinylpyrrolidone)-conjugated iridium-(III) complex (Ir-PVP) and poly(ε-caprolactone)-*b*-poly(N-vinylpyrrolidone) (PCL-PVP) nanoparticles	Iridium (III) complex	Optical imaging of tumor and metastasis
Polymer hybrid CaP nanoparticles	Tumor pH-triggered release of Mn^2+^ from CaP to boost higher contrast enhancement in hypoxic tumor regions	Mn^2+^	MR imaging of solid tumors, hypoxia and metastasis

#### Tumor-metabolite responsive NCs

In tumor cells, metabolic reprogramming changes the metabolic pathways to encounter their unquenchable craving for nutrient and energy. In this regard, tumor-associated metabolites are generally valued in estimating tumor incidence and advancement timely. Insufficient nutrient supply fluctuates cancer energy metabolism, in this case lactic acid (extracellular metabolite) declines the pH of the tumor microenvironment. Fruehauf et al. mentioned that the oxamate-functionalized NPs proficiently sequestered lactate dehydrogenase (LDH) to make an OxNP–protein complex. This effort validates a great concept for tuning NPs sensitivity via conjugation with a fundamental protein to aim a particular metabolite of cancer disease [156].

### Exogenous stimuli

When trigger stimuli are caused by external factors, such as a change in temperature, ultrasound, or light responsiveness, they are known as exogenous stimuli. In this system, contrast agents are employed to visualize NCs retention in cancer cells.

#### Temperature responsive NCs

Thermo responsive NCs use temperature changes as the trigger to release their cargo, as they can change their hydrophilic and hydrophobic balance, solubility, or structural features in response to a particular temperature. These NCs show a low critical solution temperature (LCST), below which the constituents of a mixture are misciblee [157]. Thermoresponsive polymers with an LCST are promising candidates for drug delivery, as are stimuli-responsive hydrogels that can prevent drug degradation and display rapid on/off switching [158, 159]. Hydrogels can be injected as viscous liquids and become jellified under physiological conditions. For instance, Tipa et al. [160] synthesized a biocompatible and injectable thermo-responsive hydrogel that was jellified in situ at 28 °C and injected with a 5 N force. The hydrogel contained clay NPs whose interlaminar spaces were intercalated with methylene blue (MB) for controlled release with enhanced encapsulation efficiency. Notably, the Pluronic hydrogel demonstrated controlled long-term MB release. Similarly, Ahsan et al. [161] formulated a thermosensitive chitosan-based, cross-linked injectable hydrogel for the sustained delivery of disulfiram to cancer cells as a long-term cancer therapy, and found that the drug-loaded hydrogels allowed greater cellular uptake than the free drugs.

Graphene oxide (GO) has a high ratio of surficial functional groups that permits the modification of bioactive molecules, as well has high dispersibility in aqueous conditions; however, its applications are limited by non-targeted and uncontrolled drug release. To overcome these limitations, Kazempour et al. modified GO with biodegradable and hydrophilic polymeric components, such as poly(N-vinylcaprolactam) (PNVCL) and poly(glycolic acid), which conferred pH and temperature sensitivity to allow the effective loading and release of oxaliplatin in MCF-7 cells [162]. Similarly, Farjadian et al. synthesized smart temperature-and pH-responsive NCs based on a random copolymer of poly(N-isopropylacrylamide-co-acrylamide) [163]. The presence of a hydrophilic group in acrylamide increased the LCST to 37 °C (close to body temperature) and ultimately made the structure suitable for drug delivery. This structure was further modified with hydrazine and conjugated to DOX via a Schiff base linkage (acid-cleavable bond) to make NCs responsive to heat and pH.

Given that hyperthermia increases the temperature of body tissues to as high as 113 °C and increases the sensitivity of cancer cells to chemotherapeutics, combining these two effects as thermo-chemotherapy could enhance the efficacy of cancer therapy. Mirrahimi et al. [164] developed a novel multifunctional nanocomplex, consisting of an alginate nanogel loaded with cisplatin and AuNPs, for combined therapy. In vivo thermometry studies showed that under 532 nm laser irradiation, the temperature increased and exerted a higher thermal dose due to the optical absorption characteristics of AuNPs; this finding indicated that these NCs suppressed tumor growth due to their thermo-responsive behavior.

#### Light-responsive NCs

Various NCs can respond to light and have the potential to adjust the irradiation wavelength, power, and affecting area [165]; therefore, light-responsive NCs have recently been developed for targeted controlled drug delivery. NCs can be sensitive to any form of light, including ultraviolet, visible, or near-infrared, which can also affect biological systems such as cancer cells or tumors. By controlling the range of irradiation, light-responsive tumor therapies can be precisely conducted to minimize or avoid negative effects on normal cells. Polyplexes [166], polymeric micelles [167], liposomes [168], nanogels [169], and nanorods [170] have all been exploited as light-responsive NCs using materials such as gold nanocomposites, CNTs, graphene, and organic molecules.

NCs can undergo conformational changes in response to light via the structural conversion of light-sensitive molecules. For instance, UV–Vis-responsive photoswitching NCs have exhibited potential for loading with drugs, including DOX, docetaxel, and paclitaxel, for cancer therapy [171]. However, UV–Vis has a short wavelength, which limits the application of these NCs. Therefore, NIR light-responsive NCs may be more appropriate for controlled drug delivery [172]. Fan et al. [173] synthesized photo-responsive degradable hollow mesoporous organo silica NCs for anti-cancer drug delivery. The NCs were based on singlet oxygen (^1^O_2_)-responsive bridged organoalkoxysilanes [from a 9, 10-dialkoxy-anthracene (DN)-based precursor] and wrapped with graphene oxide QDs. Upon irradiation, the these QDs generated ^1^O_2_, leading to the cleavage of ^1^O_2_-responsive bridges, NCs degradation, and the release of the anticancer drug.

#### Ultrasound-responsive NCs

High-frequency waves, named as ultrasound might affect NCs for the controlled drug release at tumor sites. Currently, it has been considerably applied in the various biomedical applications, for instance gene delivery and imaging-guided drugs. It can initiate the release of the drugs via the heat produced from cavitation phenomena [174]. The ultrasound-responsive nanocarriers might be used for tumor ultrasound imaging, which is considerably safe, cost-effective and commonly applied in clinics. The gas and perfluoropentane incorporated NCs [175], which could generate gas (e.g., CO_2_) in biological atmosphere [176], have exhibited tumor-site specific imaging at great intensity. Besides, the ultrasound-responsive property might be used for boosting the intracellular delivery of siRNA or DNA in tumors [177]. Nevertheless, the huge size of ultrasound-responsive NCs can limit their penetration within tumor tissues, due to the weak penetration [178]. In general, the drug-loaded ultrasound-sensitive NCs might be used for tumor therapy [179] as well as theranostics [180].

## Mechanisms of targeting stimuli responsive NCs to tumor site

**Fig. 7:**
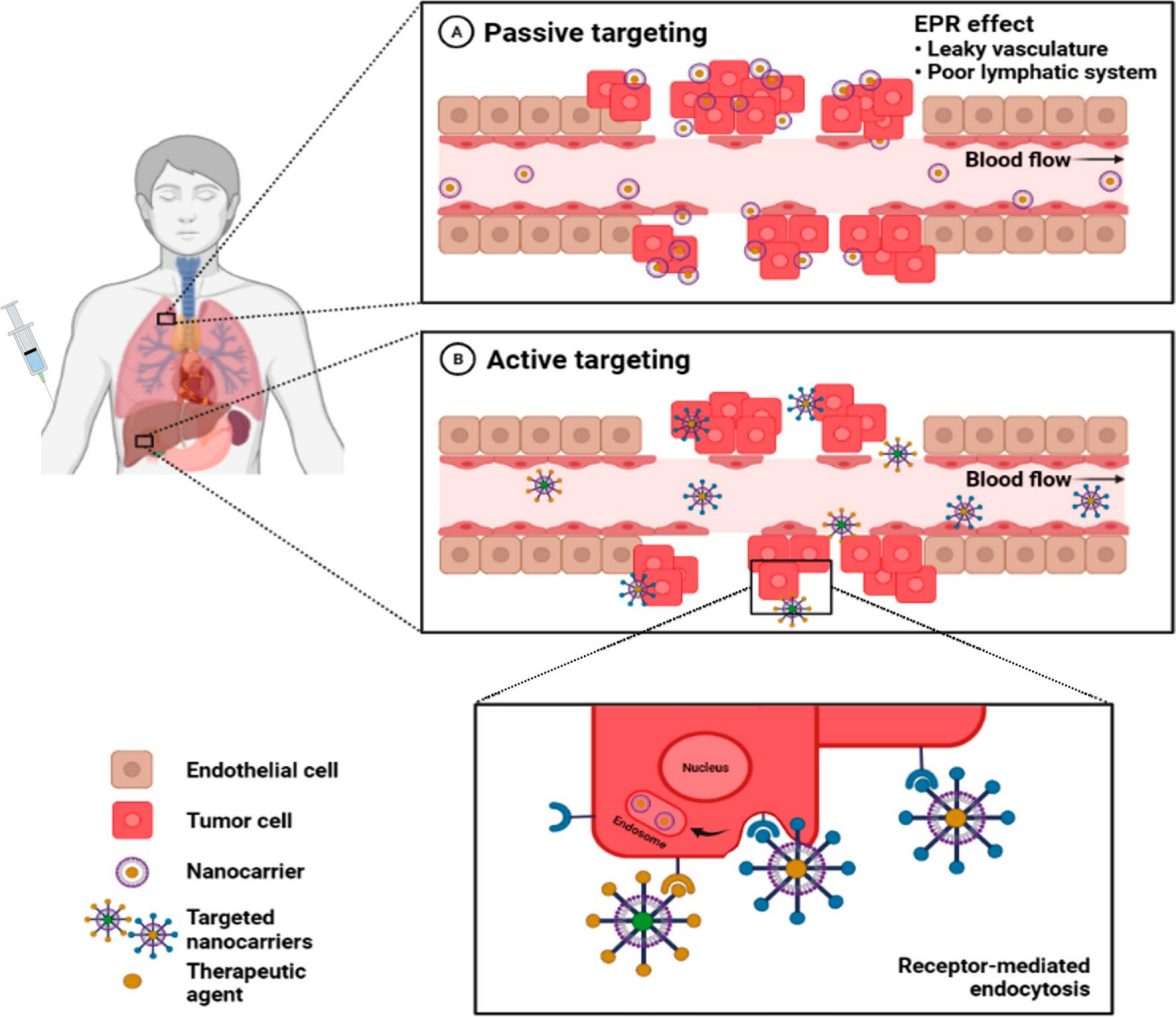
Mechanisms of targeting NCs to tumors. **A** Passive targeting by nanomedicines is due to the enhanced permeability and retention (EPR) effect, which involves their extravasation from leaky tumor vasculature and poor lymphatic drainage. **B** Active targeting is achieved by functionalizing nanomedicines with targeting ligands that recognize tumor cell receptors, which increases cell specificity and uptake. Figure has been created using Biorender

Cancer cell specificity is an important feature of NCs for drug delivery because it improves their therapeutic efficacy while sparing normal cells from damage. However, NCs can only reach cancer cells if they deceive the body's own defense system. There are two mechanisms of targeted drug delivery: passive and active targeting (Fig. 7) [181]. Both of these methods aim to prevent contact between normal cells and harmful chemicals, reduce negative dose-limiting consequences, and prevent the development of drug-resistant malignant cells.

### Passive targeting

Compared to normal cells, rapidly developing malignant cells put higher stress on the endothelium of blood vessels, resulting in the formation of new vessels or the depletion of existing vessels. During this process, pores are formed on cancer cells, allowing the adsorbed macromolecules or NCs to enter the newly formed vessels [182]. Inadequate lymphatic drainage within tumors prevents the NCs from leaving, thereby enabling them to discharge the loaded drugs into the cancer cells. This phenomenon is known as the EPR effect and is an important factor for passive targeting [125]. This effect is influenced by the diameter of NPs; studies have shown that smaller NPs have a higher permeation but do not spill into regular channels [183], whereas micro molecules are preferentially removed by immune defense systems [184]. Interstitial fluid pressure (IFP) is another obstacle to the effective deposition of drug-loaded nanocarriers in solid tumors [185]; however, successful NCs alterations can bypass biological obstacles. At first, passively targeted NCs moved the clinic several years back with the agreement of PEGylated liposomal doxorubicin (DOXIL™) [186]. There are numerous studies regarding passive targeting by lipid NPs also. For instance, sclareol-SLNs with an typical particle size of 88 nm, has disclosed considerably growth inhibition effect on lung cancer cells along with a sustained drug release when compared to the free drug alone [187]. Conjugation of curcumin with solid lipid NPs is additional report for passive targeting of osteocarcoma cancer tissues with extremely higher tissue availability [188]. In contrast, for passive targeting of melanoma and glioblastoma, temozolomide-SLN exhibited maximum inhibition in proliferation with low toxicity in normal counterparts when compared to temozolomide without solid lipid NPs [189].

A variety of NPs are now being used in clinical trials, including Genexol-PM™ in Korea and ProLindac™ and Opaxio™ in the United States [190]. Awada et al. and Burris et al. have also validated the standards and/or therapeutic efficacy of a variety of other NCs in clinical trials, including AZD2811, NK911, and CPX-1 [191, 192]. Though, many of these NCs basically change the toxicological profile, or drug solubility, some have also presented substantial survival advantages and improved therapeutic efficacy as stated in clinical reports. One case is Abraxane™ which established notably greater response rates as compared to standard paclitaxel treatment in a phase III trial conducted in breast cancer patients with metastasis [193].This findings is tremendously promising for this field which opens up innovative prospects in employing NCs as smart delivery vehicles for various drugs. Unfortunately, passive targeting has several drawbacks, including non-specific drug distribution, restriction of the EPR effect, and varying blood vessel permeability across tumors [194].

### Active targeting

Active targeting approaches are considerably more complicated than the passive approaches. It is remarkable that the active targeting is critical for the drug and gene delivery to the site of interest and thereby augments the therapeutic efficacy and restricts side effects on the normal tissues. Active targeting is able to improve the amount of drug delivered to the target tumor cell compared passively targeted NCs. Following accumulation in the tumor site, the drug competence may be even augmented by the active targeting. This is accomplished via the decoration of the NCs surfaces with ligands which could bind to overexpressed receptors in tumor cells [181]. This strategies will expand the affinities of the NCs for the tumor cell surface to improve the drug penetration capability. To ensure that the drugs are released at a specific location at a predetermined ratio, the binding of NCs ligands to cancer cell receptors causes receptor-mediated endocytosis, which allows the NCs to effectively deliver the therapeutic drugs. Consequently, active targeting is specifically tailored toward the transport of macromolecular drugs, such as proteins and siRNAs. Targeting ligands include proteins (antibodies and fragments), nucleic acids (aptamers), and other peptides, vitamins, and carbohydrates [195]. The ligands attach to receptors on targeted cells, among which the most common are the transferrin receptor, folate receptor, glycoproteins, and epidermal growth factor receptor. Bartlett et al. demonstrated that the active targeting of nucleic acids into cells may also be used to silence luciferase beacons targeting the transferrin receptor in neuroblastoma xenografts [196]. Although there are currently no commercially available active targeting NCs on the market, clinical trials are under way for NCs based on liposome-targeted and polymeric NPs. For instance, MBP-426, MCC-465, SGT53, MM-302, BIND-014, CALEA-01, cetuximad-decorated Doxil/Caelyx liposomes, and a retroviral vector are currently in phase I/II clinical studies for primary therapeutic targets, including EGF, Tf-R, PSMA, the surface of gastric cancer cells, and HER-2 [191, 192].

In general, crossing the tumor endothelium is a fundamental footstep in the expedition of NPs from the administration region to diseased tissues. Earlier literature proposed that the vascular barrier of tumor blood vessels is compromised [197]. It is believed that compared to healthy blood vessels, tumor blood vessels could demonstrate certain pathological features that affect NPs activity throughout accumulation inside tumor tissues [198] concisely, systemically controlled NPs could transport from the tumor blood vessel lumen via the endothelial spaces into the tumor interstitial area [197]. This is the central mechanism for both active and passive NPs targeting [199].

## Challenges and future prospects

As nanotechnology and innovative functional materials have advanced, so has interest in polymer and hybrid-based nanocarriers for drug delivery. Developing nanocarriers for the delivery of pharmaceuticals has long been a focus of many scientists. Despite substantial advances with hybrid nanocarriers, polymer-based nanocarriers have had a lot of success in clinical studies. While in circulation in the blood, nanocarriers must (1) prevent early leakage of therapeutic agents, (2) possess targeting ability so that therapeutic agents may aggregate at tumor sites and decrease severe side effects on healthy tissues, show biocompatibility, and (3) demonstrate degradability.

There has been a surge in interest in nanocarriers due to a variety of factors including their biodegradability, biocompatibility, physiological medium stability, and structural instability in malignancies. Polymer or hybrid-based nanocarriers with stimuli-responsive properties have required a great deal of effort to create effectively. They were able to change their pH, enzyme, thermal, and ultrasonic responsiveness to deliver medications to tumors. It is possible to deliver chemotherapy chemicals to tumors more accurately owing to nanocarriers, which both encapsulate and distribute the chemicals in a targeted manner. To improve the therapeutic efficacy of hybrid-based nanocarriers, these nanocarriers have also been used in imaging. Hybrid nanocarriers, which are being developed at a rapid pace, are anticipated to stimulate a combination of diagnostic and therapeutic in the area of cancer therapy. Researchers have proven that theragnostic hybrid nanocarriers with theragnostic capabilities may be utilized to correctly cure cancer in the future. To date, the hybrid-based nanocarriers’ therapeutic efficacy has been improved by using these nanocarriers in imaging. Nanocarriers that combine diagnostic and therapeutic capabilities are anticipated to be encouraged by the rapid development of hybrid nanocarriers. Hybrid nanocarriers with theragnostic capabilities may be employed to correctly treat cancer in the future as previously predicted.

A few issues remain in the design of nanocarriers containing stimuli-responsive polymer or hybrid materials. Although progress has been made in the development of stable nanocarriers in a healthy medium and stable nanocarriers at tumor locations, it is still challenging to accomplish. The discovery of novel hybrid-based nanocarriers, despite significant progress, continues to impede the development of nanomedicines. Nanocarriers have also been overdesigned in recent decades to integrate many functionalities into a single molecule in order to create a multifunctional nanomedicine. Nanocarriers that are too complicated to be used in clinical trials are generally the result of over-engineering. Nature is always guided by the simplest and most cost-effective rules of operation. Polymer or hybrid nanocarriers that can be synthesized economically and at large scale will thus be the most beneficial. In the end, these issues and obstacles will encourage biomedical scientists to better decipher the relationships between the nanocarriers structural and functional aspects and to enhance nanocarrier design. Consequently, experts from a variety of fields will be better able to realize the promise of stimuli-responsive nanocarriers for cancer treatment if they work together.

## Conclusion

In this review, we have provided a broad overview of various materials that can be used as drug delivery NCs for cancer therapy, as well as stimuli-based drug delivery systems and the different mechanisms of targeting. Due to their unique characteristics, clinicians have been able to use drug delivery NCs as monotherapies or as adjuncts to current treatments in order to enhance therapeutic efficacy (Table 4). Although some of these systems have failed in clinical trials, numerous new and intriguing materials are currently under development and display tremendous potential, indicating that new therapeutic alternatives may soon be available. In particular, stimuli-sensitive NCs provide high specificity as well as various desirable drug delivery functions, including regulated release, tumor accumulation, and better diagnostic and therapeutic efficacy.

**Table 4:** List of NCs used in clinics or in clinical trials [200]

Products	Drug	Nanocarrier	Application
In clinics			
ADI-PEG 20	Arginine deaminase	Polymeric	Hepatocellular carcinoma
Doxil	Doxorubicin	Polymeric	Leukemia, lymphoma, and carcinoma
AP5280	Platinum	Polymeric	Solid tumors
DepoCyt	Cytarabine	Liposomal	Lymphomatous meningitis
MAG-CPT	Camptothecin	Polymeric	Solid tumors
Visudyne	Verteporfin	Liposomal	Macular degeneration
Oncaspar	L-Asparaginase	Polymeric	Lymphoblastic leukemia
Pegasys	Interferon alfa-2a	Polymeric	Hepatitis B and hepatitis C
Clinical trials			
PNU166945	Paclitaxel	Polymeric	Solid tumors
Lipoplatin	Cisplatin	Liposomal	Non-small cell lung cancer
XMT-1001	Camptothecin	Polymeric	Gastric cancer and lung cancer
Onco-TCS	Vincristine	Liposomal	Relapsed non-Hodgkin lymphoma
OSI-211	Lurotecan	Liposomal	Head, neck, and ovarian cancer
SPI-077	Cisplatin	Liposomal	Head, lung, and neck cancer
PEG-SN38	Irinotecan derivate	Polymeric	Solid tumors and breast cancer
Livatag	Doxorubicin	Polymeric	Liver cancer
NKTR-105	Docetaxel	Polymeric	Solid tumors and ovarian cancer
Paclical	Paclitaxel	Polymeric	Breast, lung, and ovarian cancer
PEG-docetaxel	Docetaxel	Polymeric	Solid tumors

## Data Availability

Not applicable.

## Abbreviations

NCs: Nanocarriers
MDR: Multiple Drug Resistance
NPs: Nanoparticles
EPR: Enhanced Permeability and Retention
MSNCs: Mesoporous Silica NCs
AuNCs: Gold (Au) NCs
MNCs: Magnetic NCs
CNNCs: Carbon Nanotube NCs
QDs: Quantum Dots
DOX: Doxorubicin
RES: Reticulo Endothelial System
PTT: Photothermal Therapy
SPR: Surface Plasmon Resonance
PEG: PolyEthylene Glycol
FA: Folic Acid
IO: Iron Oxide
CIO: Chitosan-coated Iron Oxide
CNTs: Carbon Nanotubes
siRNAs: small interfering RNAs
MWCNTs: Multi-Walled CNTs
RF: Riboflavin
ROS: Reactive Oxygen Species
Fc: Ferrocene
TUNEL: Terminal deoxyribonucleotide transferase-mediated dUTP Nick-End-Labeling
DNR: Daunorubicin
PLGA: Poly (Lactic-co-Glycolic Acid)
PLL: Poly-L-Lysine
Tf: Transferrin
FNPs: Fibroin Nanoparticles
PEI: PolyEthyleneImine
SPARC: Secreted Protein, Acidic, and Rich in Cysteine
BSA: Bovine Serum Albumin
HSA: Human Serum Albumin
FRα: Folate Receptor alpha
BID: BH3 Interacting Domain
Bcl-2: B-cell lymphoma 2
BAX: Bcl-2-associated X
TRAIL: Tumor necrosis factor-Related Apoptosis-Inducing Ligand
CMC: Critical Micelle Concentration
RAP: Rapamycin
WOG: Wogonin
VEGF: Vascular Endothelial Growth Factor
CSCs: Cancer Stem Cells
Fab: Antibody fragment
cur: Curcumin
GSH: Glutathione SulfHydryl
COS: Chito Oligo Saccharides
LCST: Low Critical Solution Temperature
MB: Methylene Blue
GO: Graphene Oxide
PNVCL: Poly(N-VinylCaproLactam)
mio: Magnetic Iron Oxide
GOQDs: Graphene Oxide QDs
IFP: Interstitial Fluid Pressure
